# Proteomic and Metabolomic Analyses of a Tea-Tree Oil-Selected *Staphylococcus aureus* Small Colony Variant

**DOI:** 10.3390/antibiotics8040248

**Published:** 2019-12-03

**Authors:** Nathanial J. Torres, Steven D. Hartson, Janet Rogers, John E. Gustafson

**Affiliations:** Department of Biochemistry and Molecular Biology, Oklahoma State University, Stillwater, OK 74078, USA; njtorres2@usf.edu (N.J.T.); steven.hartson@okstate.edu (S.D.H.); janet.rogers@okstate.edu (J.R.)

**Keywords:** *Staphylococcus aureus*, small colony variant, tea tree oil, proteomics, metabolomics, fatty acid biosynthesis

## Abstract

Tea tree oil (TTO) is hypothesized to kill bacteria by indiscriminately denaturing membrane and protein structures. A *Staphylococcus aureus* small colony variant (SCV) selected with TTO (SH1000-TTORS-1) demonstrated slowed growth, reduced susceptibility to TTO, a diminutive cell size, and a thinned cell wall. Utilizing a proteomics and metabolomics approach, we have now revealed that the TTO-selected SCV mutant demonstrated defective fatty acid synthesis, an alteration in the expression of genes and metabolites associated with central metabolism, the induction of a general stress response, and a reduction of proteins critical for active growth and translation. SH1000-TTORS-1 also demonstrated an increase in amino acid accumulation and a decrease in sugar content. The reduction in glycolytic pathway proteins and sugar levels indicated that carbon flow through glycolysis and gluconeogenesis is reduced in SH1000-TTORS-1. The increase in amino acid accumulation coincides with the reduced production of translation-specific proteins and the induction of proteins associated with the stringent response. The decrease in sugar content likely deactivates catabolite repression and the increased amino acid pool observed in SH1000-TTORS-1 represents a potential energy and carbon source which could maintain carbon flow though the tricarboxylic acid (TCA) cycle. It is noteworthy that processes that contribute to the production of the TTO targets (proteins and membrane) are reduced in SH1000-TTORS-1. This is one of a few studies describing a mechanism that bacteria utilize to withstand the action of an antiseptic which is thought to inactivate multiple cellular targets.

## 1. Introduction

*Staphylococcus aureus* is a leading cause of hospital-acquired infections that demonstrates a propensity for acquiring resistance to antimicrobials [1]. One mechanism that enhances the ability of *S. aureus* to cause infections and tolerate antimicrobial challenge is the ability to produce small colony variants (SCVs) [2]. *S. aureus* SCVs are typically characterized by slow growth, reduced electron transport chain activity, decreased virulence factor production, reduced susceptibility to antimicrobials, and altered metabolism [3,4]. A number of transcriptional profiling and proteomic analysis studies have revealed that *S. aureus* SCVs display an altered expression of genes and proteins involved with both glycolysis and the tricarboxylic acid (TCA) cycle [5,6,7,8,9].

The SCV phenotype can result from mutations in a number of genes. Pioneering studies revealed that SCVs result from mutations in genes required for the biosynthesis of the electron transport chain components hemin and menadione (e.g., *hemB*, *hemH*, *menD*, *ctaA*) [10,11,12,13] or thymidine (e.g., *thyA*) [14]. Besides these mutations, other mutations in genes encoding a cold shock protein (*cspB*) [15], the stringent response (p)ppGpp synthetase RelA (*rsh*) [16], and an ATP-dependent helicase (*cshA*) [17], also supported SCV formation. Furthermore, the deletion of the gene encoding succinate dehydrogenase (*sdh*) also resulted in a SCV phenotype [18]. 

SCVs can also be selected with a variety of antimicrobials in the laboratory. Kanamycin-selected SCVs harbored a mutation in a chorismate biosynthetic gene (*aroD*) [19], fusidic acid-selected SCVs carried mutations in a gene that encodes ribosomal protein L6 (*rplF*) [20], and mutations in menadione biosynthetic genes (*menB* and *menH*) appear to be responsible for a H_2_O_2_-selected SCV phenotype [21].

Recently, SCVs were shown to evolve faster growth rates while maintaining aminoglycoside resistance, which suggests that infections with these organisms may pose an additional threat by maintaining antimicrobial resistance after the SCV phenotype is lost [22]. 

Tea tree oil (TTO) is a popular over-the-counter antiseptic produced from the steam distillation of the leaves and stems of tea trees, and demonstrates broad-spectrum in vitro antimicrobial activity [23,24]. TTO kills bacteria by inactivating multiple cellular targets via the denaturation of membrane and protein structures [25,26,27,28]. Challenge of *S. aureus* with TTO led to a near universal downregulation of genes encoding products required for transcription and translation, and the upregulation of genes required to maintain existing protein functions [29]. This study provided evidence to suggest that in order to survive, *S. aureus* shuts down energy-intensive metabolic pathways which become compromised during TTO challenge and attempts to maintain the function of pre-existing proteins. TTO can also select for *S. aureus* SCVs that display reduced susceptibility to TTO in addition to ethanol, isopropanol, and individual TTO antimicrobial terpenes. Compared to the parent strain, a TTO-selected SCV was also characterized by a small cell size and a thinned cell wall [30]. Genomic analysis of the TTO-selected SCV revealed the presence of two intragenic and 19 intergenic mutations [30]. One of the intragenic mutations was located in *acpP* [30], which encodes the essential acyl carrier protein (ACP) required for fatty acid biosynthesis [31]. To our knowledge, this is the first report of an antimicrobial-selected *acpP* mutation, and possibly the only reported example of an *acpP* mutation in *S. aureus*. 

Bacteria contain the fatty acid type II synthase (FASII) system in which each reaction is catalyzed by a specific enzyme with reaction intermediates carried as thioesters of ACP [32]. ACP is a critical component of FASII which is modified on a conserved serine residue (S36) by the addition of a 4′-phosphopantetheine on which fatty acid chain elongation occurs [33,34]. The initiation and elongation steps of FASII fatty acid biosynthesis consists of multiple enzymatic reactions carried out by the “Fab” proteins. The acyltransferase module of bacterial lipid biosynthesis allows for the transfer of fatty acids to glycerol-3-phosphate. To carry out these reactions *S. aureus* utilizes the PlsX/Y/C system [35]. In Gram-positive bacteria, fatty acid and phospholipid biosynthesis are regulated by the transcriptional repressor FapR which is located within the *fapR* operon that contains the following order of genes: *fapR*, *plsX*, *fabD*, *fabG,* and *acpP* [36,37]. 

In this present study, we compared the proteome and metabolome of a TTO-selected SCV and its parent strain. The alterations observed provide insight into the altered physiology exhibited by a TTO-selected SCV that supports reduced susceptibility to a substance that inactivates multiple cellular targets.

This study was presented in part at the American Society for Biochemistry and Molecular Biology Meeting 2017 (22–26 April 2017, Chicago, IL, USA).

## 2. Results

### 2.1. General Proteomics Comparison

A total of 488 proteins were identified in both SH1000 and SH1000-TTORS-1 (Table S1). In comparison to parent strain SH1000, 39 proteins were increased and 73 proteins were decreased in the SH1000-TTORS-1 proteome (Table 1). In addition, nine proteins were only detected in SH1000-TTORS-1 and three proteins were only detected in SH1000 (Table 1). Uncharacterized proteins made up a large percentage of the proteins (47/112 or 42%) altered in SH1000-TTORS-1 (Table 1). Of these, 21 were increased and 26 were decreased in relative concentrations in SH1000-TTORS-1 compared to SH1000 (Table 1). 

The relative concentrations of CspB and MenB (1,4-dihyroxy-2-naphtoyl-CoA synthase) and an additional cold shock protein (CspC) were decreased in SH1000-TTORS-1 (Table 1), and *cspB* or *menA* inactivation led to the formation of SCV mutants [15,38].

### 2.2. Alterations in Fatty Acid Biosynthesis in SH1000-TTORS-1

**Figure 1:**
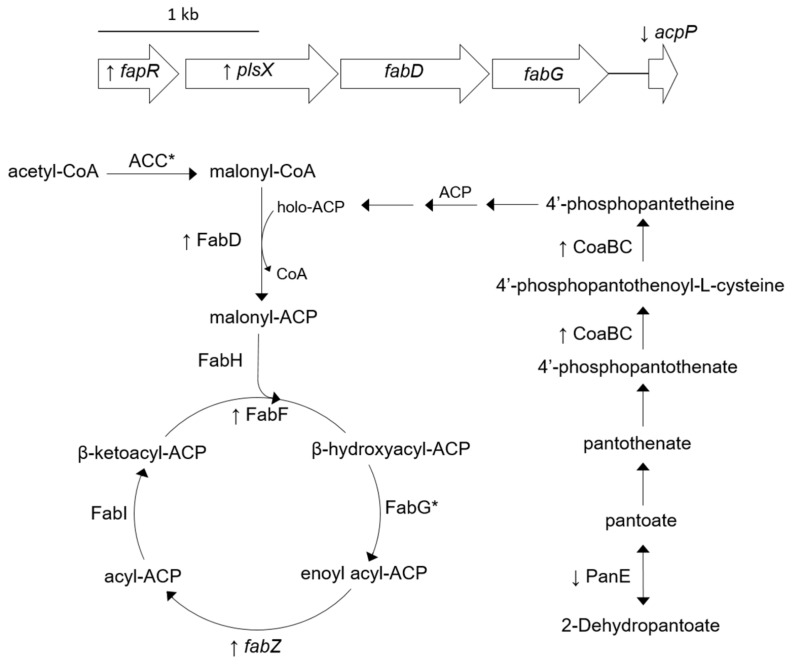
Cartoon indicating protein concentrations and gene expressions associated with fatty acid biosynthesis that are altered in SH1000-TTORS-1 compared to SH1000. Up and down arrows next to protein symbols or gene names denote increased and decreased concentration/expression in SH1000-TTORS-1 compared to SH1000. Proteins that were detected in both SH1000-TTORS-1 and SH1000 at insignificantly different (*p* ≥ 0.05) concentrations are labeled with an asterisk (*). Genes shown and the products they encode are: *fapR*, fatty acid biosynthesis transcriptional regulator; *plsX*, glycerol-3-phosphate acyltransferase; *fabD,* malonyl-CoA-acyl carrier protein transacylase; *acpP*, acyl carrier protein; and *fabZ*, 3-hydroxyacyl-acyl carrier protein dehydratase. Proteins shown are: FabD, malonyl-CoA-acyl carrier protein transacylase; *fabG*/FabG, 3-oxoacyl-acyl-carrier-protein reductase; ACP, acyl carrier protein; ACC = AccB, acetyl-CoA carboxylase biotin carboxyl carrier protein subunit, and AccC, acetyl-CoA carboxylase biotin carboxyl carrier protein subunit; FabH, β-ketoacyl-acyl carrier protein synthase; FabI, enoyl-ACP reductase; PanE, 2-dehydropantoate 2-reductase; and CoaBC, bifunctional phosphopantothenoylcysteine decarboxylase/phosphopantothenate-cysteine ligase.

Despite the fact that relative FapR concentrations are the same in SH1000 and SH1000-TTORS-1 (Table S1), RT-PCR analysis revealed that *fapR*, *plsX*, and *fabZ* were all up-regulated, while the expression of *acpP* was decreased in SH1000-TTORS-1 (Table 2, Figure 1). 

CoaBC is a bifunctional phosphopantothenoylcysteine decarboxylase/phosphopantothenate-cysteine ligase that catalyzes the final reaction in synthesizing 4′-phosphopantetheine [39,40] and PanE is a 2-dehydropantoate 2-reductase important in the biosynthesis of the 4′-phosphopantetheine precursor pantothenate [41] (Figure 1). CoaBC is increased and PanE is reduced in SH1000-TTORS-1 compared to SH1000 (Table 1, Figure 1). In addition, FabD (malonyl-CoA-ACP transacylase) and FabF (3-oxoacyl-synthase) were both increased in SH1000-TTORS-1 (Table 2, Figure 1). 

The fatty acid biosynthesis inhibitor triclosan selected SCVs that harbored mutations in one or more genes encoding FabI and FabD, a teicoplanin resistance-associated protein, and a NADH-dependent flavin oxidoreductase [42]. Triclosan-selected SCVs were also reported to produce higher concentrations of FabF, as well as FabI and FabH (β-ketoacyl-ACP synthase); however, triclosan-selected SCVs demonstrated decreased concentrations of FabD [42]. 

Uncharacterized proteins SAOUHSC_01348 and SAOUHSC_2604 were increased in relative concentrations in SH1000-TTORS-1. SAOUHSC_01348 exhibited 50% amino acid identity with the *Bacillus subtilis* acyl-CoA thioesterase YneP, that is suspected to be involved with fatty acid degradation [43]. It has been reported however that under laboratory conditions, *S. aureus* cannot degrade fatty acids [44]. SAOUHSC_02604 is an uncharacterized oxidoreductase that has a conserved FabI superfamily domain and demonstrates 26% protein identity along the entire length of the *S. aureus* enoyl-ACP-reductase FabI, which is required for fatty acid biosynthesis [45]. 

Growth of the triclosan-selected SCVs on media containing the fatty acid supplement Tween 80 led to increased colony size, which demonstrated that the SCV mutants were deficient in fatty acid biosynthesis [42]. 

The addition of Tween 80 to MHA media also led to an increase in colony size for SH1000-TTORS-1 (from 0.29 mm ± 0.05 to 0.64 mm ± 0.04, *n* = 10, *p* = 3.2 × 10^-12^), but not to the same size as the parent strain (1.45 ± 0.05 mm) which was unaffected by Tween 80-addition (1.47 ± 0.05, *n* =10, *p* = 0.48). 

### 2.3. Proteins Involved with Central Metabolism Are Altered in SH1000-TTORS-1

**Figure 2:**
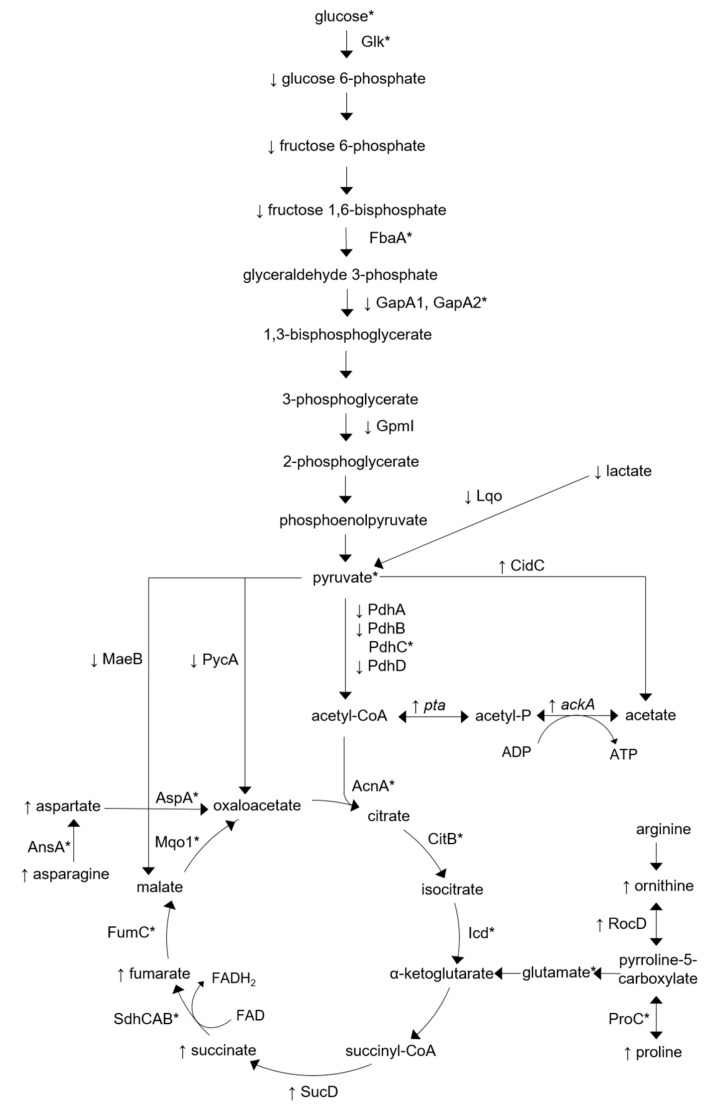
Diagram indicating protein and metabolite concentrations and gene expressions associated with central metabolism that are altered in SH1000-TTORS-1 compared to SH1000. Up and down arrows next to protein symbols, gene names or metabolites denote increased and decreased concentration/expression in SH1000-TTORS-1 compared to SH1000. Proteins and metabolites that were detected in both SH1000-TTORS-1 and SH1000 at insignificantly different (*p* ≥ 0.05) concentrations are labeled with an asterisk (*). Genes shown and the products they encode are: *pta*, phosphotransacetylase; and *ackA*, acetate kinase. Proteins shown are: Glk, glucokinase; FbaA, fructose-bisphosphate aldolase class II; GapA1, glyceraldehyde-3-phosphate dehydrogenase; GapA2, glyceraldehyde-3-phosphate dehydrogenase 2; GpmI, 2,3-bisphosphoglycerate-independent phosphoglycerate mutase; Lqo, lactate:quinone oxidoreductase; MaeB, malate dehydrogenase; PycA, pyruvate carboxylase; CidC, pyruvate oxidase; PdhA, pyruvate dehydrogenase complex, E1 component subunit alpha; PdhB, pyruvate dehydrogenase complex, E1 component subunit beta; PdhC, branched-chain alpha-keto acid dehydrogenase subunit E2; PdhD, dihydrolipoamide dehydrogenase; AcnA, citrate synthase; CitB, aconitate hydratase; Icd, isocitrate dehydrogenase; SucD, succinyl-CoA synthetase subunit alpha; SdhC, succinate dehydrogenase cytochrome β-558 subunit; Sdh, succinate dehydrogenase flavoprotein subunit; SdhB, succinate dehydrogenase iron-sulfur subunit; FumC, fumarate hydratase class II; Mqo-1, malate:quinone oxidoreductase; AnsA, L-asparaginase; AspA, uncharacterized aspartate/methionine/tyrosine aminotransferase; RocD, ornithine-oxo-acid transaminase; ProC, pyrroline-5-carboxylate reductase.

Our proteomics analysis revealed the presence of a number of proteins involved with central metabolism in both SH1000 and SH1000-TTORS-1 (Table S1) and the relative concentration of some of these proteins were altered in SH1000-TTORS-1 (Table 1, Figure 2). The glycolytic proteins glyceraldehyde-3-phosphate dehydrogenase (GapA1) and 2,3-bisphosphoglycerate-independent phosphoglycerate mutase (GpmI) were both decreased in SH1000-TTORS-1 (Table 1, Figure 2). Additional proteins that affect pyruvate metabolism (PycA, pyruvate carboxylase; MaeB, malate dehydrogenase; Lqo, L-lactate-quinone oxidoreductase; and PdhABD, pyruvate dehydrogenase complex) were also decreased in SH1000-TTORS-1 (Table 1, Figure 2). A reduction in Pdh levels in *S. aureus* SCVs has previously been reported on [8]. The pyruvate oxidase CidC which metabolizes pyruvate into acetate, was also increased in SH1000-TTORS-1 (Table 1, Figure 2). *pta* and *ackA* respectively encode a phosphotransacetylase and an acetate kinase, and the ability of Pta-AckA to metabolize acetyl-CoA into acetate is essential for *S. aureus* survival [46]. RT-PCR analysis revealed that the expression of both *pta* and *ackA* were elevated in SH1000-TTORS-1 (Table 2, Figure 2). 

Only three TCA cycle proteins were not identified in either SH1000 or SH1000-TTORS-1: SucA (2-oxoglutarate dehydrogenase E1), SucB (dihydrolipoamide succinyl-transferase), and SucC (succinyl-CoA synthetase subunit beta) (Table S1). Of the identified TCA cycle proteins, only the succinyl-CoA synthetase subunit alpha (SucD) was increased in SH1000-TTORS-1 (Table 1, Figure 2). 

### 2.4. Proteins Associated with Stress and Stringent Response Are Altered in SH1000-TTORS-1

The general stress response RNA polymerase sigma factor SigB is intimately involved with the general stress response of *S. aureus*, and plays an essential role in the formation of SCVs [47,48]. RsbU is a phosphatase that positively regulates SigB activity by releasing SigB from the anti-sigma factor RsbW [49]. The relative concentrations of both SigB and RsbU were both increased in SH1000-TTORS-1 (Table 1). In addition, a number of proteins produced by genes controlled by SigB were also altered in SH1000-TTORS-1. The genes that produce Cap5O (UDP-N-acetyl-D-mannosaminuronic acid dehydrogenase) and CidC are positively regulated by SigB [50], Cap50 was only detected in SH1000-TTORS-1, and CidC was increased in SH1000-TTORS-1 (Table 1). The genes that produce PycA, Hel (5′-nucleotidase), and SAOUHSC_02820 are negatively regulated by SigB [50], and the relative concentration of the proteins produced from these genes were decreased in SH1000-TTORS-1 (Table 1). 

The stringent response in *S. aureus* is activated when nutrients such as amino acids are limited, which results in the accumulation of the alarmone guanosine 3′, 5′-bisdiphosphate and decreased expression of genes encoding products involved with protein biosynthesis [51]. RelA is an alarmone synthetase required for the stringent response that also demonstrates guanosine 3′, 5′-bisdiphosphate hydrolysis activity [51]. *S. aureus* has two additional (p)ppGpp synthetases, RelP and RelQ, that both lack the RelA C-terminal hydrolyzing domain [52]. The relative concentration of RelQ was increased in SH1000-TTORS-1 compared to SH1000 (Table 1). This finding suggests that a component that controls the stringent response in increased in SH1000-TTORS-1, supporting the suggestion that the stringent response is partially activated in this mutant. 

Additional proteins previously reported to be reduced during the stringent response [53] were also reduced in SH1000-TTORS-1 compared to SH1000. These proteins included: PdhD, GpmI, SAOUHSC_02574 (an uncharacterized NAD/NADP octopine/nopaline dehydrogenase alpha-helical domain), TrxB (a thioredoxin reductase), and TypA (a GTP-binding protein) (Table 1). The protein SAOUHSC_02665 which encodes an uncharacterized general stress protein induced during a stringent response [53] was also increased in SH1000-TTORS-1 (Table 1). The altered expression of these proteins could be linked to the increased expression of RelQ in SH1000-TTORS-1.

### 2.5. Additional Proteins Altered in SH1000-TTORS-1

The relative concentration of a number of proteins involved with translation were decreased in SH1000-TTORS-1. These proteins included: PheT (phenylalanyl-tRNA synthetase subunit beta), TrpS (trytophanol tRNA synthetase), MetG (methionyl-tRNA synthetase), and RplY (50S ribosomal protein L25/general stress protein) (Table 1). MetG is responsible for the production of methionyl-tRNA which when formylated can be used to initiate translation [54]. The methionine aminopeptidase protein (Map) is then responsible for cleaving the N-terminal methionine following protein production [54], and the relative concentration of Map was also reduced in SH1000-TTORS-1 (Table 1). 

Two different subunits of DNA polymerase, DnaN (DNA polymerase III β-subunit) and DnaX (DNA polymerase III γ and τ subunits), and nucleotide metabolism proteins PurA (adenylosuccinate synthetase) and Tdk (thymidine kinase) were all decreased in SH1000-TTORS-1 (Table 1). The production of AtpG (F0F1 ATP synthase γ-subunit) was also decreased in SH1000-TTORS-1 and an intergenic mutation was identified between the last gene of the ATP synthase operon (*atpC*) and the hypothetical gene SAOUHSC_02339 in SH1000-TTORS-1 [30] (Table 1). 

*S. aureus* contains two copies of UDP-N-acetylglucosamine 1-carboxyvinyltransferase, MurA and MurZ, which catalyze the first committed step in peptidoglycan biosynthesis [55]. *murZ* expression is also increased in *S. aureus* exposed to peptidoglycan biosynthesis inhibitors [55]. Both MurA and MurZ were identified in the SH1000 and SH1000-TTORS-1 proteomes; however, MurZ levels were increased in SH1000-TTORS-1 (Table 1). Because SH1000-TTORS-1 demonstrated a thinned cell wall [29] and MurZ was increased in SH1000-TTORS-1, we determined the qualitative susceptibility of SH1000 and SH1000-TTORS-1 to antimicrobials that target peptidoglycan synthesis. The distance grown by SH1000-TTORS-1 on a 0 → 2 mg/L vancomycin gradient was greater (30.7 mm ± 1.15 mm, *n* = 3) than SH1000, which did not grow on this gradient at all. SH1000-TTORS-1 also grew to a greater distance on a 0 → 0.5 mg/L oxacillin gradient (52.7 mm ± 2.52 mm) compared to SH1000 (19.3 mm ± 1.15 mm, *n* = 3, *p* < 0.05). 

### 2.6. Metabolomics Analysis

A total of 105 metabolites were identified in both strains: 16 amines and polyamines, 29 amino acids, 38 polar organic acids, and 22 sugars (Table S2). Out of these metabolites, 35 metabolites were found to be significantly altered in SH1000-TTORS-1 compared to SH1000, three metabolites were only identified in SH1000, and four metabolites were only identified in SH1000-TTORS-1 (Table 3; Table 4).

Eleven amino acids were found in higher relative concentrations (Table 3) and 10 sugars were found in lower concentrations (Table 4) in SH1000-TTORS-1 compared to SH1000. 

Asparagine and aspartic acid are increased in SH1000-TTORS-1 and during the degradation of these amino acids they can enter the TCA cycle after being turned into oxaloacetate [56] (Figure 2), and aspartic acid is one of eight amino acids that is initially degraded by *S. aureus* during growth in media without a preferred carbon and energy source [56]. The relative concentration of RocD which is involved with ornithine and proline biosynthesis [57] was increased in SH1000-TTORS-1 (Table 1, Figure 2), and both ornithine and proline are increased in SH1000-TTORS-1 (Table 3, Figure 2). The increased proline accumulation could be attributed to the increased relative concentration of the proline/choline/glycine betaine transporter OpuD [58] in SH1000-TTORS-1 (Table 1). Ornithine is synthesized from arginine during the urea cycle and is a precursor needed in the synthesis of glutamate and proline (Figure 2) [57]. Both proline and ornithine can be metabolized into glutamate which can then enter the TCA cycle as α-ketogluterate (Figure 2). 

The concentration of glucose 6-phosphate, fructose 6-phosphate, and fructose 1,6-bisphosphate were decreased in SH1000-TTORS-1 compared to SH1000 (Figure 2, Table 3) indicating that gluconeogenesis or sugar accumulation is reduced in this mutant. 

The concentrations of TCA cycle intermediates fumarate and succinate were both increased in SH1000-TTORS-1 which occurs in conjunction with increased SucD expression (Table 1; Table 3). 

Lactate is synthesized from pyruvate in the absence of oxygen via lactate dehydrogenase [59] and lactate concentrations were decreased in SH1000-TTORS-1, yet there was no difference in the D-lactate dehydrogenase levels in SH1000-TTORS-1 and SH1000 (Table S1). In conjunction with the reduced lactate level in SH1000-TTORS-1, we note that the relative concentration of a protein (Lqo) which is responsible for metabolizing lactate into pyruvate, was also decreased (Figure 2) [60]. 

The only sugar found to be increased in SH1000-TTORS-1 and not detected in SH1000 was N-acetylglucosamine (Table 3) which is a precursor for peptidoglycan biosynthesis [61]. 

The reduction in PurA (adenylosuccinate synthetase) observed in SH1000-TTORS-1 (Table 1), that occurs with a concomitant reduction in adenosine and guanosine, was not even detected in this mutant (Table 3).

## 3. Discussion

SH1000-TTORS-1 harbors a mutation in *acpP* [30], which leads to the production of an ACP with an amino acid alteration (A34D) that is 2 amino acids away from the highly conserved S36 that is modified with a 4′-phosphopantetheine moiety that acts as the site for fatty acid attachment during fatty acid elongation [34]. We now report that SH1000-TTORS-1 exhibits a number of other alterations that further supports the hypothesis that fatty acid biosynthesis in this mutant is defective (Figure 1). SH1000-TTORS-1 demonstrated the altered expression of genes required for the fatty acid and lipid biosynthesis (*plsX*, *fabZ*, and *acpP*) and the control of this process (*fapR*). SH1000-TTORS-1 also expressed an increased quantity of CoaBC and reduced quantity of PanE, which implied that the production of holo-ACP is altered in this mutant. Furthermore, the relative concentration of FabD (malonyl-CoA-ACP transacylase) and FabF (3-oxoacyl-synthase) (Figure 1) and two uncharacterized proteins (SAOUHSC_01348, SAOUHSC_02604) possibly involved with fatty acid biosynthesis and degradation, were all increased in SH1000-TTORS-1. Lastly, similar to triclosan-selected SCVs [42], growth of SH1000-TTORS-1 with the fatty acid supplement tween-80 led to an increase in colony size. We previously reported that the overall fatty acid content of SH1000 and SH1000-TTORS-1 were similar [30], so we propose that fatty acid biosynthesis is defective and slowed in SH1000-TTORS-1. 

Similar to previously characterized *S. aureus* SCVs [5,6,7,8,9], SH1000-TTORS-1 also demonstrated alterations in central metabolism. The relative concentration of GapA1 and Gpm1 which are central to glycolysis and gluconeogenesis were reduced in SH1000-TTORS-1. Six proteins involved in pyruvate metabolism were also reduced in SH1000-TTORS-1. The defect in fatty acid biosynthesis observed in SH1000-TTORS-1 probably also contributes to altered acetyl-CoA metabolism. Collectively, this data implies that the flow of pyruvate and acetyl-CoA into the TCA cycle is altered in SH1000-TTORS-1. At the same time, the relative concentration of CidC and *ackA* and *pta* expression were increased in SH1000-TTORS-1, which indicated an alteration in acetate metabolism. ATP production via the Pta/AckA pathway is thought to be important for growth when glucose is not abundant [56]. Collectively, these findings indicate that pyruvate, acetyl-CoA and acetate metabolism are skewed within SH1000-TTORS-1. Surprisingly, metabolomic analysis revealed that the concentrations of pyruvic acid in both SH1000 and SH1000-TTORS-1, were not significantly different (Table S2). 

In SH1000-TTORS-1 SucD, succinate, and fumarate, were all increased in SH1000-TTORS-1 indicating increased flow of carbon in this part of the TCA cycle in SH1000-TTORS-1. During carbon catabolite repression (CCR) in bacteria, the presence of preferred carbon sources leads to the repression of genes whose products are involved in the catabolism of non-preferred carbon sources [62]. *S. aureus* can readily degrade a number of amino acids and it was suggested that glutamate and amino acids that generate glutamate, particularly proline, serve as the major carbon source in defined media lacking a preferred carbon source [56]. We propose that the reduction in sugar content in SH1000-TTORS-1 alleviates carbon catabolite repression (CCR) allowing for the catabolism of amino acids (ornithine, proline, asparagine and aspartate), which are provided from the increased amino acid pool observed in SH1000-TTORS-1, to produce TCA intermediates and energy (Figure 2). 

It has been previously reported that the production of ribosomal proteins can be altered in *S. aureus* SCVs [8] and an *S. aureus* SCV mutant harboring a *relA* mutation demonstrated increased expression of TCA cycle enzymes [16]. Protein synthesis and the expression of tRNA synthetases are also reduced during the stringent response [51]. SH1000-TTORS-1 demonstrated reduced production of proteins essential to translation, including tRNA-amino acid synthetases, increased RelQ production and the altered production of proteins affected by the stringent response. The increase in free amino acid pools in SH1000-TTORS-1 therefore likely results from downturned protein production and the activation of an aberrant stringent response.

The increased production of SigB and RsbU, and the altered expression of SigB-controlled proteins demonstrates that the general stress response is activated in SH1000-TTORS-1. Considering the altered metabolic state of SH1000-TTORS-1 overall, it is not surprising that SigB and RsbU are increased in an effort to deal with the stress this mutant is experiencing and it should be noted that SigB is required for SCV formation [47,48].

The thinned cell wall [30], increased expression of MurZ, increased N-acetylglucosamine content, and alteration in cell wall antimicrobial susceptibility reported in this study supports the notion that peptidoglycan biosynthesis is also compromised in SH1000-TTORS-1. Furthermore, the reduction in DNA polymerase III, the major DNA replicative complex of the cell, indicates that DNA replication is reduced in SH1000-TTORS-1. This reduction in adenosine and guanosine is likely linked to a reduced DNA replication and ATP production brought on by the slowed growth and the altered metabolism observed in this mutant. 

## 4. Materials and Methods

### 4.1. Strains, Antibiotic Gradient Plate Analysis, Colony Size, and Chemicals

The strains employed in this study included methicillin-susceptible strain SH1000 [63] and a TTO-selected SCV, SH1000-TTORS-1 [30]. Mueller Hinton broth (MHB), Luria broth (LB), and bacteriological-grade agar were purchased from Becton Dickinson and Company (Franklin Lakes, NJ). All chemicals were purchased from Sigma Aldrich Co. (St. Louis, MO), unless otherwise noted. TTO utilized for this study was purchased from Aura Cacia (Urbana IA, *Melaleuca alternifolia* product code A191139) and contained 44.1% terpinen-4-ol and 3.9% α-terpineol (v/v) which complies with the TTO International Standard (ISO 4730).

Gradient plate analysis was performed with MHB overnight cultures and Mueller Hinton agar (MHA) as previously described [64], and confluent growth along the gradient was measured in mm ± standard deviation (*n* = 3). Colony size determination was carried out as previously described [29] by diluting overnight MHB cultures and plating on MHA and MHA containing 0.1% (v/v) Tween 80. Random colonies were then measured for each strain grown on both media using a caliper in mm ± standard deviation (*n* = 10). 

### 4.2. Protein Extraction

Total proteins were isolated from *S. aureus* strains SH1000 and SH1000-TTORS-1 in triplicate following the procedure of Wolff et. al. 2008 [65]. Briefly, overnight cultures were used to inoculate 250 mL of LB (initial OD_580_ = 0.01) which were then incubated (37 °C, 200 rpm) until the cultures reached an OD_580_ = 0.7. The cells were then pelleted (8000× *g*, 10 min, 4 °C) and stored at −80 °C. These cell pellets were then thawed and washed twice with TBS buffer (50 mM Tris-HCl, 150 mM NaCl, pH 7.4) and re-suspended in 1 mL of lysis buffer (50 mM Tris-HCl, 150 mM NaCl, 1 mM EDTA, 1 mM DTT, 1 mM PMSF, pH = 7.4). These suspensions were then loaded into a 2-mL cryovial tube containing 1 g of 0.1 mm glass beads (Biospec Products, Bartlesville OK) and the cells were lysed by agitation using a Mini-Beadbeater (Biospec Products) (5 × 50 sec @ 4200 rpm) with 2-min resting periods on ice in between agitation cycles. The cell lysates were then pelleted (8000× *g*, 10 min, 4 °C) twice to remove whole cells and glass beads, and sodium azide was added (final concentration of 0.02% v/v) before samples were stored at −80 °C. Protein concentrations were determined using the Bradford method [66] using bovine serum albumin (Bio-rad, Hercules CA) as a standard. 

### 4.3. Mass Spectrometry Analysis

Each protein sample was analyzed using two replicate LC-MS/MS analyses on a hybrid LTQ-Orbitrap mass spectrometer (LC-MS/MS) (Thermo Fisher Scientific, Waltham, MA) as previously described [67]. Proteins were identified by using the Andromeda application with MaxQuant [68,69] to search the MS data against a database of *S. aureus* proteins downloaded from Uniprot (*S. aureus* strain NCTC 8325, ID 9306). Alterations in protein expression were quantified on the basis of peptide peak intensities, via the LFQ algorithm [70] embedded in MaxQuant v. 1.5.3.8. Statistically significant differences in protein levels were calculated via the *t*-test algorithms embedded in Perseus 1.5.3.2 ([71,72]; Max Planck Institute of Biochemistry). A two-fold alteration in protein concentration and a Student’s *t*-test *p*-value of < 0.05 was used as the threshold criteria for significant differences in protein expression (Table 1).

### 4.4. Metabolite Extraction and Analysis

Metabolites were extracted from both SH1000 and SH1000-TTORS-1 in triplicate as previously described [73]. Briefly, overnight cultures were used to inoculate 125 mL of LB (initial OD_580_ = 0.01) and incubated (37 °C, 200 rpm) until an OD_580_ = 0.07 was reached. Cells from each sample were then collected by centrifugation (10,000× *g*, 5 min, 4 °C) and washed once with 1× PBS (137 mM NaCl, 2.7 mM KCl, 10 mM Na_2_HPO_4_, 2 mM KH_2_PO_4_, pH = 7.4) and re-pelleted (10,000× *g*, 5 min, 4 °C). Metabolic quenching was achieved by adding 500 μL of cold methanol (−20 °C) and all samples were stored at −80 °C. Metabolite analysis was carried out by the Roy J. Carver Biotechnology Center, University of Illinois at Urbana-Champaign, Urbana, IL, USA as previously described [73]. Metabolite relative concentrations were normalized using 100 mg of dry cell weight and statistically significant alterations in metabolite levels were based upon a Student’s *t*-test *p*-value < 0.05 and reported as the average ± standard error of the mean for all three biological replicates.

### 4.5. RNA Extraction and RT-PCR

Initially, exponentially-grown 50-mL LB cultures (see above) were pelleted (8000 rpm, 4 °C, 10 min), and re-suspended in 1 mL of TRIzol (Ambion-Thermo Fisher Scientific, Waltham, MA). Cell lysis was completed using a single cycle of bead beating as described above. The resulting supernatant was then extracted with chloroform (1/5 the total volume) with agitation, and then centrifuged (12,000× *g*, 4 °C, 15 min). The aqueous layer was then placed in a fresh microfuge tube and the RNA was then precipitated with ice cold isopropanol (2.5 × the total volume) on ice, followed by centrifugation (12,000× *g*, 4 °C, 20 min). The resulting RNA pellet was then washed with 70% ethanol, re-pelleted (7500× *g*, 4 °C, 10 min) and air-dried. All RNA samples were treated with the DNA-free Kit (Ambion-Thermo Fischer Scientific, Waltham, MA) and cDNA samples were produced utilizing SuperScript III Reverse Transcriptase per the manufacturer’s instructions (Invitrogen-Thermo Scientific, Waltham, MA).

cDNA samples were then interrogated by real-time PCR using the LightCycler 96 Real-Time PCR system (Roche, Indianapolis, IN) and FastStart Universal SYBR Green Master (ROX) (Roche). Gene-specific primers used in the RT-PCR analysis are found in Table S3. Critical threshold values were normalized using 16S rRNA and expression values were calculated using the 2^−ΔΔCt^ method [74].

## 5. Conclusions

Overall this study provides additional insight into a mechanism that a bacterial pathogen utilizes to thwart the action of a popular “natural” antiseptic that inactivates multiple targets. We propose that the slowed or reduced production of the general targets of TTO (proteins and membranes) and altered metabolism allow SH1000-TTORS-1 to withstand the antimicrobial action of TTO better than the parent strain. This reduction in major biosynthetic pathway activity, stress response activation, and overall alteration in metabolism also likely contributes to the reduced growth and small colony phenotype observed in SH1000-TTORS-1.

## Figures and Tables

**Table 1:** Proteins altered in SH1000-TTORS-1 compared to SH1000 (*p* ≤ 0.05), and those only detected in SH1000-TTORS-1 or SH1000.

^a^ Locus ID	Protein Symbol	Description	Functional Category	Log2-Fold Change
**Increased in SH1000-TTORS-1 compared to SH1000**				
SAOUHSC_00356	YkoI	membrane protein	uncharacterized	2.1
SAOUHSC_00452	YaaQ	cyclic-di-AMP receptor	uncharacterized	1.4
SAOUHSC_00468		reactive intermediate/imine deaminase	uncharacterized	1.5
SAOUHSC_00604	WrbA	multimeric flavodoxin	uncharacterized	2.5
SAOUHSC_00833		nitroreductase	uncharacterized	1.9
SAOUHSC_00850		SUF system FeS assembly protein	uncharacterized	1.6
SAOUHSC_00894	RocD	ornithine-oxo-acid transaminase	amino acid metabolism	1.8
SAOUHSC_00921	FabF	3-Oxoacyl-synthase	lipid metabolism	1.8
SAOUHSC_00942	RelQ	GTP pyrophosphokinase	nucleotide metabolism	1.4
SAOUHSC_01178	CoaBC	bifunctional phosphopantothenoylcysteine decarboxylase/phosphopantothenate-cysteine ligase	cofactors and secondary metabolites	1.3
SAOUHSC_01186	PrpC	serine/threonine protein phosphatase	uncharacterized	1.1
SAOUHSC_01198	FabD	malonyl CoA-acyl carrier protein transacylase	lipid metabolism	3.0
SAOUHSC_01218	SucD	succinyl-CoA synthetase subunit alpha	carbohydrate metabolism	4.0
SAOUHSC_01261	CinA	competence-damage inducible protein	stress response	2.6
SAOUHSC_01264		71 aa protein	uncharacterized	18.4
SAOUHSC_01346	OpuD	proline/choline/glycine betaine transporter	transporter	3.0
SAOUHSC_01348	YneP	acyl-CoA thioesterase	uncharacterized	6.1
SAOUHSC_01401	LysA	diaminopimelate decarboxylase	amino acid metabolism	1.2
SAOUHSC_01490	Hup	DNA-binding protein HU	DNA replication, recombination and repair	1.3
SAOUHSC_01594		predicted oxidoreductase	uncharacterized	1.9
SAOUHSC_01768	Tag	3-methyladenine DNA glycosylase	DNA replication, recombination and repair	1.7
SAOUHSC_01814		universal stress protein	uncharacterized	1.4
SAOUHSC_02029		phi ETA orf 56-like protein	uncharacterized	1.3
SAOUHSC_02150		thioredoxin	uncharacterized	2.5
SAOUHSC_02298	SigB	RNA polymerase sigma factor	stress response	1.3
SAOUHSC_02301	RsbU	SigmaB regulation protein	stress response	1.7
SAOUHSC_02365	MurZ	UDP-N-acetylglucosamine 1-carboxyvinyltransferase	cell wall metabolism	1.2
SAOUHSC_02370		acetyltranferase GNAT family	uncharacterized	2.1
SAOUHSC_02390		lytic regulatory protein	uncharacterized	1.7
SAOUHSC_02447	CurA	NADPH-dependent curcumin reductase	uncharacterized	5.7
SAOUHSC_02604		oxidoreductase	uncharacterized	9.2
SAOUHSC_02626		139 aa protein	uncharacterized	1.3
SAOUHSC_02665		general stress protein 26	uncharacterized	2.1
SAOUHSC_02689	YndB	conserved protein	uncharacterized	1.8
SAOUHSC_02737		epimerase/dehydratase	uncharacterized	1.7
SAOUHSC_02774		alkylhydroperoxidase family enzyme	uncharacterized	3.5
SAOUHSC_02827		GNAT superfamily predicted acetyltransferase	uncharacterized	2.6
SAOUHSC_02849	CidC	pyruvate oxidase	carbohydrate metabolism	1.8
SAOUHSC_02980		cysteine hydrolase	uncharacterized	1.4
**Decreased in SH1000-TTORS-1 compared to SH1000**				
SAOUHSC_00002	DnaN	DNA polymerase III subunit beta	DNA replication, recombination and repair	−1.4
SAOUHSC_00019	PurA	adenylosuccinate synthetase	nucleotide metabolism	−1.4
SAOUHSC_00074		periplasmic binding protein	transporter	−2.6
SAOUHSC_00200	PrsW	membrane proteinase	uncharacterized	−1.7
SAOUHSC_00204	Hmp	globin domain-containing protein	uncharacterized	−1.4
SAOUHSC_00284	Hel	5′-Nucleotidase	nucleotide metabolism	−3.4
SAOUHSC_00362		208 aa protein	uncharacterized	−1.9
SAOUHSC_00369		prolipoprotein diacylglyceryl transferase	uncharacterized	−1.7
SAOUHSC_00417		esterase/lipase	uncharacterized	−2.2
SAOUHSC_00442	DnaX	DNA polymerase III subunits gamma and tau	DNA replication, recombination and repair	−1.6
SAOUHSC_00461	MetG	methionyl-tRNA synthetase	protein synthesis	−1.3
SAOUHSC_00474	RplY	50S ribosomal protein L25/general stress protein Ctc	protein synthesis	−1.5
SAOUHSC_00483		133 aa protein	uncharacterized	−1.5
SAOUHSC_00513		23S rRNA (guanosine(2251)-2′-O)-methyltransferase RlmB	protein synthesis	−1.6
SAOUHSC_00542		phosphatase	uncharacterized	−1.5
SAOUHSC_00578	MvaD	mevalonate diphosphate decarboxylase	cofactors and secondary metabolites	−1.2
SAOUHSC_00634	MntC	ABC transporter substrate binding protein	transporter	−2.5
SAOUHSC_00637	MntA	uncharacterized ABC-type Mn2+/Zn2+ transport system, ATPase component	transporter	−2.3
SAOUHSC_00669	YkaA	conserved protein	uncharacterized	−1.5
SAOUHSC_00717		electron transfer DM13	uncharacterized	−3.0
SAOUHSC_00785	TrxB	thioredoxin reductase	amino acid metabolism	−1.8
SAOUHSC_00795	GapA1	glyceraldehyde-3-phosphate dehydrogenase	carbohydrate metabolism	−1.6
SAOUHSC_00798	GpmI	2,3-Bisphosphogylcerate-independent phosphoglycerate mutase	carbohydrate metabolism	−1.6
SAOUHSC_00819	CspC	cold shock protein	stress response	−4.6
SAOUHSC_00838	YwqG	292 aa protein	uncharacterized	−1.9
SAOUHSC_00933	TrpS	tryptophanyl-tRNA synthetase	protein synthesis	−1.2
SAOUHSC_00985	MenB	1,4-dihydroxy-2-naphthoyl-CoA synthase	cofactors and secondary metabolites	−1.4
SAOUHSC_01040	PdhA	pyruvate dehydrogenase complex, E1 subunit alpha	carbohydrate metabolism	−1.5
SAOUHSC_01041	PdhB	pyruvate dehydrogenase complex, E1 subunit beta	carbohydrate metabolism	−1.5
SAOUHSC_01043	PdhD	dihydrolipoamide dehydrogenase	carbohydrate metabolism	−1.3
SAOUHSC_01058	TypA	GTP-binding protein	unclassified	−1.7
SAOUHSC_01064	PycA	pyruvate carboxylase	carbohydrate metabolism	−1.7
SAOUHSC_01091	SpoU	tRNA G18 (ribose-2′-O)-methylase	uncharacterized	−1.4
SAOUHSC_01093	PheT	phenylalanyl-tRNA synthetase subunit beta	protein synthesis	−1.4
SAOUHSC_01110	Ecb	extracellular complement-binding protein	virulence factor	−4.3
SAOUHSC_01163		RluA family pseudouridine synthase	uncharacterized	−1.7
SAOUHSC_01488		heptaprenyl diphosphate synthase subunit 1	uncharacterized	−1.3
SAOUHSC_01525		phage tail tape measure protein	uncharacterized	−2.2
SAOUHSC_01587	RluB	ribosomal large subunit pseudouridine synthase B	protein synthesis	−1.3
SAOUHSC_01661		tRNA A22 N-methylase	uncharacterized	−1.6
SAOUHSC_01679	MiaB	tRNA A37 methylthiotransferase	uncharacterized	−1.8
SAOUHSC_01698	YbhY	RNA-binding protein	uncharacterized	−1.3
SAOUHSC_01716		PrtC family collagenase-like protease	uncharacterized	−2.7
SAOUHSC_01735		tRNA A37 threonylcarbamoyladenosine dehydratase	uncharacterized	−1.6
SAOUHSC_01810	MaeB	malate dehydrogenase	carbohydrate metabolism	−2.0
SAOUHSC_01858		phenylalanyl-tRNA synthetase subunit beta	uncharacterized	−1.5
SAOUHSC_01901	Tal	transaldolase	carbohydrate metabolism	−1.4
SAOUHSC_01979		DNA-binding transcriptional regulator	uncharacterized	−2.0
SAOUHSC_02096		91 aa protein	uncharacterized	−1.6
SAOUHSC_02102	Map	methionine aminopeptidase	protein fate	−1.8
SAOUHSC_02133	PncB	nicotinate phosphoribosyltransferase	cofactors and secondary metabolites	−1.3
SAOUHSC_02139		pyrazinamidase/nicotinamidase	cofactors and secondary metabolites	−1.5
SAOUHSC_02258		fatty acid repression mutant protein/oxidoreductase	uncharacterized	−2.1
SAOUHSC_02268	ScrB	sucrose-6-phosphate hydrolase	carbohydrate metabolism	−1.3
SAOUHSC_02343	AtpG	F0F1 ATP synthase subunit gamma	energy metabolism	−1.3
SAOUHSC_02360	Tdk	thymidine kinase	nucleotide metabolism	−2.0
SAOUHSC_02525	AcrB	multidrug efflux pump subunit	uncharacterized	−1.4
SAOUHSC_02544	MoaB	molybdopterin precursor biosynthesis	cofactors and secondary metabolites	−1.6
SAOUHSC_02549	ModA	molybdenum ABC transporter substrate-binding protein	transporter	−1.7
SAOUHSC_02574		NAD/NADP octopine/nopaline Dehydrogenase, alpha-helical domain	uncharacterized	−1.6
SAOUHSC_02627		Acetyl esterase/lipase	Uncharacterized	−1.9
SAOUHSC_02652		YhdH/YhfP family putative quinone oxidoreductase	Uncharacterized	−1.3
SAOUHSC_02697	YecC	amino acid ABC transporter ATP-binding protein	transporter	−1.4
SAOUHSC_02699	FliY	substrate binding domain of ABC transporters involved in cystine import	transporter	−1.5
SAOUHSC_02739	PanE	2-dehydropantoate 2-reductase	cofactors and secondary metabolites	−1.9
SAOUHSC_02767	NikA	peptide ABC transporter substrate binding protein	transporter	−2.2
SAOUHSC_02791		pyrophosphohydrolase	DNA replication, recombination and repair	−1.5
SAOUHSC_02820		ATP-binding cassette domain of the bacitracin-resistance transporter	uncharacterized	−14.1
SAOUHSC_02834	SrtA	sortase	protein fate	−1.7
SAOUHSC_02911		adenine nucleotide alpha hydrolase superfamily predicted ATPase	uncharacterized	−4.5
SAOUHSC_02927	Lqo	lacatate:quinone oxidoreductase	carbohydrate metabolism	−1.7
SAOUHSC_02976	ManA	mannose-6-phosphate isomerase	carbohydrate metabolism	−2.0
SAOUHSC_03045	CspB	cold shock protein	stress response	−4.8
**Proteins only detected in SH1000-TTORS-1**				
SAOUHSC_00077		diderophore synthetase component	uncharacterized	
SAOUHSC_00128	Cap5O	cap5O protein/UDP-N-acetyl-D- mannosaminuronic acid dehydrogenase	virulence factor	
SAOUHSC_00668	VraG	ABC transporter permease	transporter	
SAOUHSC_01279		hydrolase alpha/beta fold domain-containing protein	uncharacterized	
SAOUHSC_01258		protein of unknown function	uncharacterized	
SAOUHSC_02460		aldo/keto reductase	uncharacterized	
SAOUHSC_02866	YdfJ	membrane protein	uncharacterized	
SAOUHSC_02908		fructosamine-3-kinase	uncharacterized	
SAOUHSC_02935	GbsR	DNA binding transcriptional regulator	stress response	
Proteins only detected in SH1000				
SAOUHSC_00197		glutaryl-CoA dehydrogenase	lipid metabolism	
SAOUHSC_01128	ArgF	ornithine carbamoyltransferase	amino acid metabolism	
SAOUHSC_02648		L-lactate permease	transporter	

**^a^** Numbers represent strain NCTC 8325 locus IDs.

**Table 2:** Comparison of SH1000-TTORS-1- and SH1000-specific gene expression measured by RT-qPCR.

Locus ID	Gene Symbol	Function	Fold-Change in Gene Expression in SH1000-TTORS-1
SAOUHSC_00574	*pta*	Phosphate acetyltransferase	2.7
SAOUHSC_01196	*fapR*	Fatty acid biosynthesis transcriptional regulator	6.3
SAOUHSC_01197	*plsX*	Glycerol-3-phosphate acyltransferase	2.3
SAOUHSC_01201	*acpP*	Acyl carrier protein	−2.0
SAOUHSC_01820	*ackA*	Acetate kinase	3.2
SAOUHSC_02336	*fabZ*	3-Hydroxyacyl-(acyl carrier protein) dehydratase	9.1

**Table 3:** Metabolites increased (*p* ≤ 0.05) in SH1000-TTORS-1 compared to SH1000.

Metabolite Relative Concentration Per Gram Dry Weight (Mean ± SE)				
Metabolite Class	Metabolite	SH1000	SH1000-TTORS-1	Fold Increase SH1000-TTORS-1/SH1000
Amino acids	3-Hydroxyproline	*N.D.	18.9 ± 1.0	
	Asparagine	2698.8 ± 211.5	38,678.1 ± 4325.2	14.3
	Aspartate	120,734.5 ± 3382.8	193,201.1 ± 19,069.7	1.6
	Homoserine	12.1 ± 3.6	40.9 ± 4.3	3.3
	Lysine	531.8 ± 236.9	1928.6 ± 79.5	3.6
	Methyl-lysine	1163.5 ± 140.8	1830 ± 94.7	1.6
	N-acetyltryptophan	343.9 ± 18.5	945.1 ± 157.5	2.7
	ornithine	46.1 ± 3.5	1058.9 ± 269.4	23.0
	Phenylalanine	3017.4 ± 501.1	6954.5 ± 1149.9	2.3
	Proline	7579.6 ± 515.8	20,362 ± 2232.6	2.7
	Tyrosine	578 ± 39.3	1073.2 ± 78.1	1.9
Polar organic acids	2-Hydroxyglutaric acid	35.2 ± 7.6	75.4 ± 9.3	2.1
	3-Hydroxypropanoic acid	N.D.	78.05 ± 15.7	
	Fumarate	44.1 ± 4.2	63.8 ± 0.8	1.4
	Malonic acid	N.D.	7.8 ± 2.3	
	Succinate	431.7 ± 43.7	735.6 ± 72.9	1.7
Sugars	N-acetylglucosamine	N.D.	124.1 ± 15.5	

*N.D., not detected.

**Table 4:** Metabolites decreased (*p* ≤ 0.05) in SH1000-TTORS-1 compared to SH1000.

Metabolite Relative Concentration Per Gram Dry Weight (Mean ± SE)				
Metabolite Class	Metabolite	SH1000	SH1000-TTORS-1	Fold Decrease SH1000/SH1000-TTORS-1
Amines and polyamines	Adenosine	2358 ± 110.1	1251.9 ± 102	−1.9
	Guanosine	1051.8 ± 127.9	*N.D.	
	Lactamide	46.2 ± 5.3	6.8 ± 2.3	−6.8
Amino acids	Cysteine	130.4 ± 17.1	61.2 ± 3.1	−2.1
	Glycine	8735.5 ± 164.5	5519.1 ± 613.2	−1.6
	N-acetyl-L-serine	3464.6 ± 323.1	482.4 ± 49.4	−7.2
Polar organic acids	2,4-Dihydroxybutaonic acid	116.0 ± 3.2	37.1 ± 3.4	−3.1
	2-Methyl-2,3-dihydroxypropanoic acid	1588.6 ± 90.0	389.4 ± 50.1	−4.1
	Aminomalonic acid	240.0 ± 5.7	118.4 ± 18.2	−2.0
	Gluconic acid	195.4 ± 31.7	27.3 ± 3.7	−7.2
	Glucuronic acid-6-phosphate	191.7 ± 73.3	N.D.	
	Glycolic acid	1430.8 ± 61.4	374.2 ± 52.7	−3.8
	Lactic acid	15,550.8 ± 896.5	9663.5 ± 1296.1	−1.6
	N-acetylneuraminic acid	1295.1 ± 113.2	307.9 ± 95.8	−4.2
	Oxamic acid	93.7 ± 19.6	9 ± 7.1	−10.4
Sugars	Fructose-1,6-bisphosphate	657.3 ± 61.7	262.4 ± 53.8	−2.5
	Fructose-6-phosphate	1422.4 ± 271.7	523.5 ± 44.6	−2.7
	Glucose-6-phosphate	3539.3 ± 770.5	485.5 ± 63.2	−7.3
	Glyceraldehyde	3.3 ± 1.1	N.D.	
	Mannitol	17,749.6 ± 749.9	8271.7 ± 941.2	−2.1
	Mannitol-6-phosphate	36,039.4 ± 347.6	16,171 ± 3376.65	−2.2
	Mannose	831.2 ± 70.4	74.7 ± 14.2	−11.1
	Sedoheptulose	138.9 ± 11.8	67.8 ± 14.2	−2.0
	Sedoheptulose-7-phosphate	722.7 ± 122	186 ± 25.3	−3.9
	Trehalose	3243.3 ± 892.6	127.6 ± 13.2	−25.4

*N.D., not detected.

## Supplementary Materials

The following are available online at https://www.mdpi.com/2079-6382/8/4/248/s1. Table S1. All proteins identified in SH1000 and SH1000-TTORS-1. Protein values are reported as the average log2 transformation of each protein’s peak intensity (*n* = 3). Table S2. All metabolites (per 100 mg dry weight) detected in SH1000 and SH1000-TTORS-1, *n* = 3. Table S3. Primers used for RT-PCR analysis in SH1000-TTORS-1.
