# Supplementary material for: Proteomic and Metabolomic Analyses of a Tea-Tree Oil-Selected Staphylococcus aureus Small Colony Variant
Source: Antibiotics (Basel). 2019 Dec 3;8(4):248. doi: 10.3390/antibiotics8040248 (PMC6963719; doi:10.3390/antibiotics8040248)
Supplement: Supplementary file 1 [file antibiotics-08-00248-s001.zip › supplementary/SupplementaryTableS3.docx]

Table S3. Primers used for RT-PCR analysis in SH1000-TTORS-1

RT16s-F *16s rRNA* TGAGTGCAGAAGAGGAAAGTG

RT16s-R *16s rRNA* CGTCAGTTACAGACCAGAAAGT

RTpta-F *pta* AGGTAAAGCGACTGAAGAACAA

RTpta-R *pta* TGTGCTGCACCACTAACTAAA

RTfapR-F *fapR* CGTGGTCATGTGCTGTTTG

RTfapR-R *fapR* GCTTCTGCTCTTACCGTATCA

RTplxX-F *plsX* GATGAGCCTGTTAGAGCGATTA

RTplsX-R *plsX* TTACCTGCTGACACACATCC

RTfabG-F *fabG* GACTTGCTGAGAACGGCTATAA

RTfabG-R *fabG* GACAAGCACTTTAACGCCAAG

RTacpP-F *acpP* CGTTTAGGTGTAGACGCTGATAA

RTacpP-R *acpP* CCAAACTCGTCTTCTAATTCCATTAC

RTackA-F *ackA* CAAAGAGCGGCAGAAATGTTAG

RTackA-R *ackA*  TGGTGTGAAGCCCATTGAT

RTfabZ-R *fabZ* AACAGGTGCGGTAGCTATTT

RTfabZ-R *fabZ*  CACCAGGTACTACTTGACGTTTA
